# Same‐day mastectomy and axillary lymph node dissection is safe for most patients with breast cancer

**DOI:** 10.1002/jso.26799

**Published:** 2022-01-20

**Authors:** Anselm Tamminen, Tuomo Meretoja, Ilkka Koskivuo

**Affiliations:** ^1^ Department of Plastic and General Surgery, Turku University Hospital University of Turku Turku Finland; ^2^ Breast Surgery Unit, Comprehensive Cancer Center Helsinki University Hospital and University of Helsinki Helsinki Finland

**Keywords:** complications, day surgery, mastectomy, safety

## Abstract

**Background and Objective:**

The aim of this study was to evaluate the safety of same‐day mastectomy, with or without a sentinel node biopsy (SNB) and/or axillary lymph node dissection (ALND).

**Methods:**

In this retrospective study, we reviewed 913 consecutive women who underwent a simple mastectomy for breast cancer between the years 2014 and 2019 and were treated either with same‐day surgery (SDS) or an overnight stay (OS) regime. We reviewed all surgical complications, any unplanned return to care (RTC) and the rehospitalization rate for 30 postoperative days.

**Results:**

A total of 259 patients (28%) were treated with SDS and 654 patients (72%) with an OS regime. There was no difference in RTC (odds ratio: 0.79 [95% confidence interval: 0.53–1.18], *p* = 0.26) or any major complications between the groups. None of the investigated subgroups, such as patients with previous neoadjuvant therapy, diabetes, obesity (up to a body mass index of 40 kg/m^2^), the American Society of Anaesthesiologist Class of 3, or elderly patients aged 75–84 years, showed an increased complication rate when treated with the SDS regime.

**Conclusion:**

A same‐day simple mastectomy is safe with SNB and/or ALND. It can be performed safely for most patients with stable co‐morbidities.

## 1 INTRODUCTION

Breast cancer is the most common cancer among women, and approximately one in eight women in Western countries will develop breast cancer during their lifetime.^1^ Breast cancer surgery has become increasingly conservative, which has made the treatment less cumbersome for the patients.^2^, ^3^, ^4^ Although breast conserving surgery (BCS) has become more utilized, mastectomy is still frequently needed when the patient has a large tumour compared to the breast size, the tumour is multicentric, or for the patients not eligible to receive radiation therapy.^5^

The very first mastectomies with a same‐day surgery approach (SDS) were performed in the 1980s, and some of the early studies suggested that this would lead to an increased rehospitalization rate.^6^, ^7^ In North America, SDS was especially criticized as being performed as a “drive‐through” procedure to save cost at the expense of treatment quality.^7^, ^8^, ^9^ Nevertheless, SDS was progressively utilized in North America in the 1990s.^6^, ^9^ In Europe, mastectomy has been considered more burdensome for the patient, and it has, therefore, been more frequently performed as an inpatient procedure, whereas BCS has been performed with an outpatient regime.^10^, ^11^, ^12^ Later research has shown increasing evidence of safety of the SDS with higher psychological satisfaction when compared to inpatient mastectomy.^13^, ^14^, ^15^, ^16^, ^17^ However, there is a lack of knowledge on which patients the SDS can be performed safely, and the patient selection has based more on tradition than research on the subject.^18^ Some studies have found patients having axillary lymph node dissection (ALND) to have more pain and therefore requiring admission.^13^, ^19^ Elderly patients and patients having substantial comorbidities have usually been excluded from the research. Therefore, there is a limited amount of evidence considering the safety of SDS in many subgroups with relatively high number of patients.^17^ In addition, most published research on the subject has been conducted in the late 1990s and early 2000s. Since then, the surgical approach, anaesthesiology, and outpatient surgery in general have developed. A present day research has therefore been needed to define how SDS is compatible with current treatment protocols, and which patients are eligible to undergo SDS.^20^

The aim of this study was to investigate the safety of SDS compared with overnight stay (OS) approach.

## 2 MATERIALS AND METHODS

### 2.1 Patients

All breast cancer patients having a simple mastectomy without immediate breast reconstruction (IBR) in our unit between the years 2014 and 2019 and being treated with an SDS or OS regime were reviewed in this retrospective study.

Patients undergoing mastectomy combined with or without any axillary procedure (sentinel node biopsy [SNB] and/or ALND) were included. Patient information was acquired from the Auria Clinical Informatics Register and from the patient records of Turku University Hospital. All data that was collected are listed in Appendix 1. All surgical procedure codes were reviewed and patients undergoing any concurrent non‐breast major surgery were excluded. The patients requiring prolonged postoperative hospitalization were briefly reviewed to define the reason for hospitalization but were not included in the study. Male patients were excluded.

The research protocol of the study was approved by the Turku University Central Hospital (T218/2019).

### 2.2 Patient selection for SDS

In our centre the SDS, including mastectomy with or without axillary surgery, was introduced in the summer of 2013. The criteria for the SDS were:

(1) the patient is eligible for SDS if they have a stable general health and comorbidies
(2) age <85 years
(3) the patient is willing to be discharged on the operation day
(4) an available adult companion to collect the patient from the hospital and to accompany them for the first postoperative night
(5) the operation is scheduled to be finished before 2 p.m.

The operation was scheduled, and the discharge setting was planned when the referral to the surgical unit was processed and thus before the patient had the preoperative admittance. A preliminary decision of SDS was therefore based on the information recorded in the referral, and the patient fulfilling the requirements was ensured on the preoperative admittance. Eligibility for the SDS was evaluated individually in relation to the SDS criteria. Patient selection criteria were in line with the current national recommendations considering criteria for SDS, stating that the same‐day discharge should be the primary discharge setting and that advanced age, obesity or concomitant diseases treated properly should not be considered as an obstacle for SDS.^21^

### 2.3 Perioperative protocol

SNB was performed with a triple technique: preoperative lymphoscintigraphy with ^99m^Tc nanocolloid, the perioperative use of blue dye and a hand‐held gamma probe. The frozen section study of excised sentinel nodes and immediate ALND for sentinel‐positive patients were used routinely in all patients until 2018, but only in selected cases after 2018 according to the updated guidelines.

Since 2016 all patients had a preoperative single‐dose intravenous antibiotic prophylaxis. Until 2016 the prophylaxis had been given to most patients, but not systematically.

Patients having anticoagulant therapy were instructed to continue for the perioperative period if there was no specific reason to discontinue it.

Mastectomy was performed with a SonoSurg® ultrasonic instrument (Olympus Medical Instruments, Tokyo, Japan) with few exceptions. A single drain was applied (two for bilateral procedures). The patients were given the instructions on handling the drain after the operation before they were discharged.

Patients planned for SDS were discharged if the following discharge criteria were fulfilled:

(1) Stable vital signs
(2) Normal orientation to space and time and patient is able to mobilise in a normal manner
(3) No nausea or vomiting and ability to consume food and water
(4) Ability to pass urine
(5) No sign of acute complications
(6) Presence of an adult companion.

The presumption was to discharge admitted patients in the first postoperative day. The patients unfit to be discharged were resettled into a primary health care ward for further treatment.

### 2.4 Postoperative protocol

The drain was removed, when the amount of exudation was less than 80 ml/day, but not earlier than 4 and no later than 7 days postoperatively. Afterwards, the patients were directed to primary health care for seroma punctations if needed. The pain medication prescribed was paracetamol 1 g up to three times a day or ibuprofen 400–800 mg up to three times a day. Opiates were not prescribed and few patients needing more pain medication than prescribed were admitted to the surgical ward. A postoperative check‐up control was instructed 2–3 weeks after the operation, and patients were given the control date and time when being discharged after the surgery. Patients were given contact information to the surgical unit in the case of any concerns.

### 2.5 Complication data collection

Information of any deviation from the normal course was collected from the electronic registers. The patient records for any unplanned return to care (RTC) for 30 postoperative days were evaluated. Information of RTC, antibiotic recipes, postoperative complication diagnosis (T81 in ICD‐10) or any infection registered in the Hospital Districts Antibiotic and Infection Register (SAI) were acquired from Auria Clinical Informatics Register. Laboratory test information was collected, and any given blood transfusions or taken bacterial culture samples (purulent drainage, blood) were recorded.

### 2.6 Treatment protocol for surgical site infections

SSI's requiring hospitalization were treated with antibiotics (cefuroxime 1.5* *g ×3 intravenously being the drug‐of‐choice) and, if necessary, repeated ultrasound‐guided punctures to drain the purulent exudate. Incisions were assertively avoided in order not to encounter wound healing problems.

All SSI diagnoses were re‐evaluated on the base of the CDC (Centers for Disease Control and Prevention) criteria.^22^ In case there was no described clinical manifestations of SSI and laboratory test did not refer to SSI (no elevation in CRP or leucocyte count and bacterial culture proved later negative), the diagnosis was not considered to be SSI.

### 2.7 Statistical analysis

The data were analysed using JMP 15 Pro (SAS Institute Cary) analysis software. The form of the information was evaluated with a statistician and the statistical analysis was preplanned in the data collection phase.

Univariate analysis was performed to compare patient characteristics between groups and bivariate analysis to identify predictors of postoperative complications. All complications were individually compared with all patient and operation related variables (Appendix 1). A chi‐square test was performed for the categorical variables and a two‐sample *t* test for those normally distributed and a Wilcoxon test for nonnormally distributed continuous variables. The variables having a relationship *p* < 0.15 were qualified with multivariable logistic regression analysis (Step 1). Variables including less than five cases were not considered eligible for statistical analysis, but clinically relevant, such variables were combined for the analysis. All factors qualifying Step 1 were cross evaluated to each other to eliminate correlating variables (Step 2). If significant correlation was found, the clinically less meaningful variable was removed, if it was possible to define (Step 3). In logistic regression, we disqualified the variable having the highest *p* value one by one until only statistically significant variables (*p* < 0.05) remained (Step 4). After multivariate analysis, the variables removed from the analysis in Step 3 were reevaluated to ensure no faulty exclusion was made (step 5). The procedure was repeated with the discharge regime factor (SDS vs. OS) included in the analysis and the results were compared to ensure that similar results were acquired from both methods (Step 6). Finally, we repeated the analysis for all subgroups including a minimum of 10 patients in both the SDS and OS groups to detect any subgroup having divergent results. Based on the subgroup analysis, it could be concluded whether the subgroup would or would not be eligible for outpatient mastectomy (Step 7). As a result, the odds ratio (OR) for any complication in the SDS group versus OS group and for all individual subgroups was defined.

## 3 RESULTS

A total of 913 patients underwent a simple mastectomy and were discharged either on the day of operation or on the next day. In total 259 patients underwent the operation in SDS regime, and 654 patients underwent the operation in OS regime. The patient characteristics and the information of concurrent operations performed with the mastectomy, previous BCS's and the changes in the rate of SDS by year are shown in Table 1.

**Table 1:** Patient characteristics

	Same‐day surgery	Overnight stay	*p* value
Number of patients	259 (28%)	654 (72%)	
Age, years (mean, IQR)	61 (49–67)	68 (58–77.25)	<0.001
BMI, kg/m^2^ (mean, IQR)	25.5 (22.7–29)	26.3 (23.1–29.7)	0.11
ASA Class			
ASA I	58 (22%)	78 (12%)	<0.001
ASA II	162 (63%)	305 (47%)	
ASA III	39 (15%)	257 (39%)	
ASA IV	0 (0%)	14 (2.1%)	
Diabetes	11 (4.2%)	70 (11%)	0.003
Smoking status			0.24
Smoker	46 (18%)	90 (14%)	
Nonsmoker	206 (79%)	527 (80%)	
Not known	7 (3%)	37 (6%)	
History of ipsilateral BCS and radiation therapy	16 (6.2%)	33 (5.0%)	0.49
Neoadjuvant therapy	30 (12%)	66 (10%)	0.51
Year of operation			<0.001
2014	28 (19%)	121 (81%)	
2015	29 (22%)	105 (78%)	
2016	49 (31%)	110 (69%)	
2017	35 (25%)	105 (75%)	
2018	50 (32%)	107 (68%)	
2019	68 (39%)	106 (61%)	
Axillary procedure			<0.001
None	2 (0.7%)	13 (2.0%)	
Operated previously	52 (20%)	54 (8.3%)	
SNB only	79 (31%)	226 (35%)	
ALND	126 (49%)	361 (55%)	
Bilateral breast cancer and bilateral surgery	4 (1.5%)	35 (5.4%)	0.01
Symmetry procedure on contralateral side	10 (3.9%)	53 (8.1%)	0.02
Mastectomy as a reoperation after BCS	45 (17%)	47 (7.2%)	>0.001

*Note*: Data expressed as *n* (%) unless otherwise specified.

Abbreviations: ANLD, axillary lymph node dissection; ASA, American Society of Anaesthesiologists; BCS, breast conserving surgery; BMI, body mass index; IQR, interquartile range; SNB, sentinel lymph node biopsy.

The patients demanding more than one night's hospitalization were reviewed, and it was discovered that they were considerably older (median age 84 years, interquartile range: 78–89 years) and they had more comorbidities (88% with ASA Classes 3–4) than the patients operated in the SDS or OS regime. These patients were not examined in more detail.

Of the patients preoperatively planned to be treated with the SDS regime, 59 (19%) were admitted for an OS. None of the patients had to be admitted due to acute surgical complications: 24 patients (41%) had to be admitted for scheduling reasons (operation finishing later than 2 p.m.), three patients (5%) reported that their postoperative pain was not under control (using the numeric pain rating scale, NPRS >4) and 32 patients (54%) had social reasons, usually not having the required care‐taker.

No patients were admitted due to nausea, bleeding, or impaired alertness.

The 59 patients having an unplanned admittance were evaluated in detail. The patients did not differ from the other patients in the SDS group (mean age 59 years, body mass index [BMI] 25.6 kg/m^2^, ASA Class I 16 patients (27%), ASA Class II 31 patients (53%) and ASA Class III 12 patients (20%). The number of patients who underwent SNB + ALND was slightly higher in this subgroup (37 patients, 63%). The number of complications in this subgroup did not differ from the other patients in SDS or OS groups. There was in total 11 RTC's (19%), one reoperation (1.7%), three rehospitalizations (5.1%) and three SSI's (5.1%).

Number of postoperative events and related risk factors are shown in Table 2. There was no 30‐day mortality. All local SSI's were successfully treated with antibiotics and repeated punctures to remove the drainage. No surgical interventions were required. One patient (41 years, good health in general) in the SDS regime had a septic infection on the first postoperative day, leading to a disseminated intravascular coagulopathy, a long intensive care unit episode and bilateral femoral amputation, but the patient did survive.

**Table 2:** Summary of complications in the 30 postoperative days

Complication	Same‐day surgery	Overnight stay	Odds ratio (SDS vs. OS)	95% CI	*p* value
Any RTC	40 (15%)	131 (20%)	0.79	0.53–1.18	0.26
Rehospitalization	12 (4.6%)	32 (4.9%)	1.09	0.54–2.20	0.81
Reoperation for complications	3 (1.6%)	11 (1.7%)	1.12	0.27–4.67	0.87
Surgical site infection	13 (5.0%)	37 (5.7%)	0.86	0.45–1.67	0.66
		38			
Unplanned return to ED	39 (15%)	125 (19%)	0.66	0.43–1.00	0.05
for infection	12 (4.6%)	33 (5.0%)	0.88	0.71–1.72	0.71
for seroma punctation	17 (6.6%)	41 (6.3%)	0.83	0.44–1.58	0.57
for any other surgery related issue^a^	7 (2.7%)	44 (6.8%)	0.39	0.17–0.87	0.021*
Admission regarding another speciality	3 (1.1%)^b^	7 (1.1%)^c^	1.08	0.28–4.22	0.91

*Note*: If reasonable, the category having less than five events were combined to reach adequate quantity for statistical evaluation.

Abbreviations: CI, confidence interval; ED, emergency department; OS, overnight stay; RTC, unplanned return to care; SDS, same‐day surgery.

* Statistical significance *p* < 0.05.

^a^ Wound dehiscence or other problems with wound healing, drainage issues, surgical site pain.

^b^ Including nonspecific interstitial pneumonia, pneumonia, infection of unknown origin after the initiation of chemotherapy.

^c^ Including transient ischemic attack (2), pyelonephritis, diabetic hyperglycaemia, atrial fibrillation, distal radius fracture, bradycardia after too high betablocker dosage.

**Figure 1:**
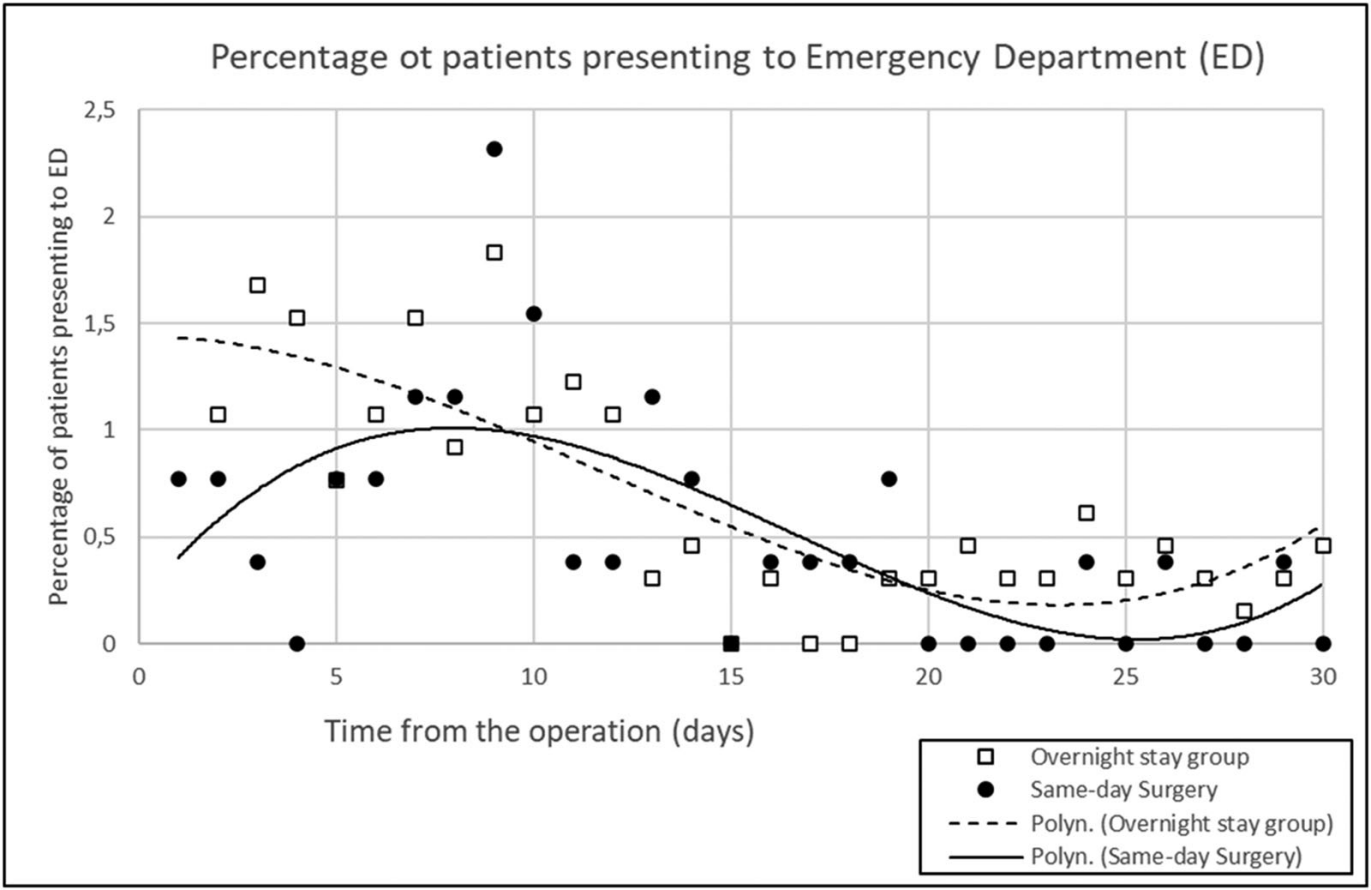
The percentage of patients presenting to the Emergency department in respect of time from the surgery. Trend line is a fitted polynomic function of 3rd degree. The number of patients presenting to the Emergency department is higher in the Overnight Stay group for 1 week after the surgery, but after that the difference seems to disappear

In total, 40 patients (15%) had RTC in the SDS group and 131 (20%) in the OS group. The timing of the RTC in each group is presented in Figure 1. It is seen that the rate of RTC is higher in OS group in the first days after the operation, but the difference is absent after the first postoperative week.

OR for SDS versus OS for all individual complications are shown in Table 2. OR for analysed subgroups are shown in Table 3.

**Table 3:** Odds ratio for RTC (SDS vs. OS) in all patient subgroups reviewed

Patient group (number of patients)	Odds ratio for any RTC (SDS vs. OS)	95% Confidence interval	*p* value
Age of 75–84 years (195)	0.20	0.03–1.52	0.12
BMI 30–35 (136)	0.57	0.21–1.53	0.27
BMI 35–40 (48)	0.40	0.07–2.36	0.31
ASA I (136)	0.48	0.18–1.34	0.16
ASA II (467)	0.93	0.56–1.55	0.78
ASA III (296)	0.47	0.15–1.43	0.18
Mastectomy as a reoperation after BCS with positive margins (92)	1.05	0.25–4.47	0.95
History of ipsilateral BCS and radiation therapy (49)	2.04	0.43–11.90	0.43
Surgeon's experience			
Up to 20 mastectomies (64)	0.20	0.02–1.72	0.14
21–50 (116)	0.21	0.07–1.77	0.21
51–100 (124)	1.11	0.43–2.86	0.83
Over 100 (605)	0.86	0.53–1.40	0.55
Axillary procedure			
SNB (305)	0.40	0.16–0.99	0.049*
ALND (487)	0.85	0.52–1.40	0.53
Bilateral procedure (102)	1.43	0.26–7.96	0.68
Diabetes (81)	0.89	0.17–4.58	0.89
Smoker (current or former) (289)	0.89	0.49–1.60	0.69
Neoadjuvant therapy (96)	0.96	0.33–2.79	0.94

*Note*: *Statistical significance *p *< 0.05.

Abbreviations: ALND, axillary lymph node dissection; ASA, American Society of Anaesthesiologists; BCS, breast conserving surgery; BMI, body‐mass index; OS, overnight stay surgery; RTC, unplanned return to care; SDS, same‐day surgery; SNB, sentinel node biopsy.

OR for any RTC in the SDS group was 0.79 (95% confidence interval [CI]: 0.53–1.18, *p* = 0.26). Variables showing statistical significance for any RTC were (1) high BMI, (2) history of ipsilateral breast cancer treated with BCS and RT and (3) ALND. There was no correlation with (1) older age, (2) neoadjuvant therapy, (3) mastectomy being a reoperation for BCS or (4) the experience of the surgeon.

In the subgroup analysis, statistical significance was reached only in the SNB group, in which the SDS group had less RTC (OR 0.40, CI: 0.16–0.99, *p* = 0.049).

## 4 DISCUSSION

This study did not show increase in any major complications in the SDS group. None of the 17 investigated subgroups was found to be unsuitable for SDS mastectomy. Interestingly, the rate of RTC was lower in SDS than OS regime in most subgroups, including patients aged 75–84 years, ASA Class levels 1–3, obese patients with BMI up to 40 kg/m^2^ and patients having a reoperation, although the statistical significance was not reached in any of these groups. There was one group (mastectomy + SNB) with less RTC in the SDS group (*p* = 0.049). However, considering the high number of subgroups investigated, this is supposed to be an incidental statistical finding rather than a true evidence of association.

The highest BMIs in the SDS group were up to 45 kg/m^2^ with no complications, but the number of high BMI patients was too low to perform the statistical analysis. Minor postoperative problems, including wound healing problems demanding only topical treatment, were more frequent in the OS group.

The SDS and OS groups were different in respect to patient age, ASA Class and proportion of diabetic patients. Diabetes was not shown to be a risk factor for any of the complications. Patients with a high ASA Class were in higher risk of having a reoperation, which there were only a few. Age was not a risk factor for any complication except seroma punctations, which were more frequent in younger patients.

There was an increasing tendency for the use of SDS during the study period. This was not an intended change, nor was there any change in the SDS criteria. The proportion of patients treated in SDS regime increased likewise in all investigated subgroups. Presumably, the good experience from the SDS resulted in a more determined utilization of SDS.

The number of patients having an unplanned admittance in the SDS group was rather high (59/318, 19%). This group of patients was examined in detail. The patients in this subgroup did not differ from the other patients in the SDS group. The number of complications was deficient to include this group as an individual group in the statistical analysis, but it is seen that the number of complications is similar to the rest of the OS group and excluding this subgroup from the analysis would not change the results. This is an expected finding, considering that the unplanned admittance was not due to medical reasons such as surgical complications, but rather due to scheduling or social reasons, which should not be associated with increased number of complications. This was, however, important finding to be verified since it could be assumed, that this subgroup would differ from the other patients in the study.

The rate of major complications is in accordance with previous literature. There are several studies showing no increase in complication risk after SDS mastectomy.^11^ Marla et al.^10^ performed a systematic review of the studies, and they concluded that SDS was safe, did not increase the risk of complications and engendered high patient satisfaction. Keehn et al.^16^ showed that there was no difference in unexpected returns to the ED or readmissions to the hospital between SDS patients and those admitted for overnight. Vuong et al.^23^ showed no increase in the ED admittance or rate of reoperations or readmissions. Warren et al.^6^ showed nearly equal rates of complications and rehospitalization even in elderly women when comparing SDS and OS groups. Cordeiro et al.^24^ showed in their large analysis of more than 40 000 patients, that the patients treated with inpatient regime had more complications than those treated as outpatients (OR: 1.37, 95% CI: 1.16–1.63, *p* = 0.004).

Importantly, none of these studies are randomized clinical trials. Some of the studies were performed comparing patients before and after the implementation of the SDS, and such studies have not shown any increase in the complication rate after the implementation of the SDS.^11^, ^23^

The previous literature suggests that SDS has certain psychological benefits. Marchal et al.^19^ stated that women having SDS tend to experience less side effects and are more satisfied with the procedure than the patients treated with an inpatient regime. Shahbazi et al.^20^ stated that SDS is largely influenced by patient expectation. Dooley et al.^8^ reported that patients undergoing SDS felt having more control over their treatment and recovery and therefore SDS could result in faster recovery and more effective and complete psychological adjustment. McManus et al.^25^ found that SDS patients had a high level of satisfaction and experienced faster healing and recovery. Margolese et al.^15^ found that SDS patients manifest a significantly better emotional adjustment and fewer psychological distress symptoms.

Referring to the previous literature, we suppose that the result of OS having more ED presentations for minor postoperative problems could be related to the psychological factors. We do not expect this to be the only explanation as it is possible, for example, that there were more solitary patients in the OS group, which could be associated with a higher rate of RTC.

In many previous studies there have been wide demographic difference between the SDS and OS groups. Even when this is taken into account in the statistical analysis, this may lead to flawed results. Although we also had differences between the SDS and OS groups, the differences were smaller than in many previous studies, and we consider the groups somewhat comparable to each other. Considering the retrospective nature of the study and inevitable selection of the patient groups, we consider the differences acceptable. We suppose that reaching substantially higher similarity between the groups would require a prospective randomized study.

## 5 LIMITATIONS OF THE STUDY

The main limitation with this study was its retrospective nature. Retrospectively, we cannot exclude the possibility of patient selection to the SDS or OS groups being influenced by some unspecified factor not included in the study.

We did not have information concerning the patients' social circumstances, especially whether the patients were solitary or whether they had a spouse or a family. In total 19% of the patients who were planned to have SDS were admitted overnight, and in majority of the cases (54%) the reason was a lack of care‐taker. Social circumstances could be related to the rate of RTC especially in minor concerns. The topic would be an interesting subject for further research.

## 6 CONCLUSION

Same‐day mastectomy is a feasible option for most patients with breast cancer, when IBR is not planned. In this study, none of the patient groups investigated were found to have an increased risk of complications when treated with SDS regime. On the contrary, minor problems requiring RTC were more frequent in the OS group.

## FUNDING INFORMATION

The study was supported by grants from the Foundation of Turku University Hospital and University of Turku.

## CONFLICT OF INTERESTS

The authors declare that there are no conflict of interests.

## SYNOPSIS

Breast cancer is the most common cancer worldwide and mastectomy is frequently required operation. The study shows that mastectomy can be safely performed on a same‐day basis even for most patients with stable comorbidities.

## Supporting information

Supporting information.

## Data Availability

The data that support the findings of this study are available on request from the corresponding author. The data are not publicly available due to privacy or ethical restrictions.
