# Supplementary material for: Same‐day mastectomy and axillary lymph node dissection is safe for most patients with breast cancer
Source: J Surg Oncol. 2022 Jan 20;125(5):831–8. doi: 10.1002/jso.26799 (PMC9303414; doi:10.1002/jso.26799)
Supplement: Supplementary file 1 — Supporting information. [file JSO-125-831-s001.doc]

| Patient related | Surgery related | Adverse Events (30 day) |
| --- | --- | --- |
| Age (years) | Operation day | **Any unplanned return to care (RTC)** |
| American Society of Anesthesiologists (ASA) Class | Discharge day | Any reoperation for complication |
| Previous breast surgeries | Surgeon´s experience | Any presentation to ED |
| History of breast cancer and radiation therapy | Mastectomy being a re-operation for breast conserving surgery (BCS) | Any rehospitalization |
| Smoking status | Axillary procedure | Postoperative haematoma |
| Neoadjuvant therapy | Bilateral surgery | Blood transfusion |
| Diabetes | Duration of surgery (min) | Wound dehiscence and/or skin flap necrosis |
| Weight (kg) | Blood loss (ml) | Surgical Site Infection |
| Height (cm) | Pain score after operation | Seroma |
| Body Mass Index (BMI) | Postoperative nausea | Drainage issues |
|  | Use of antibiotic prophylaxis |  |

Appendix 1. The data collected for statistical analysis.
